# Leptin Levels and Bone Mineral Density: A Friend or a Foe for Bone Loss? A Systematic Review of the Association Between Leptin Levels and Low Bone Mineral Density

**DOI:** 10.3390/ijms26052066

**Published:** 2025-02-27

**Authors:** Dina Cosme, Ana Cordeiro Gomes

**Affiliations:** 1 i3S—Instituto de Investigação e Inovação em Saúde, Universidade do Porto, 4200-135 Porto, Portugal; 2 Serviço de Medicina Interna, Unidade de Saúde Local São João, 4200-319 Porto, Portugal

**Keywords:** leptin, bone mineral density, bone loss, cystic fibrosis bone disease

## Abstract

The introduction of CFTR modulators in the clinics has improved body mass index in cystic fibrosis (CF) individuals. Leptin is a major regulator of appetite and energy expenditure but is also involved in bone metabolism. Whether circulating leptin levels are associated with low bone mineral density (BMD) and fracture risk in CF remains unknown. Therefore, the present study aims to analyze and integrate the current evidence linking leptin and bone loss in CF. As no scientific evidence was found, we focused on secondary dysregulations of bone loss in CF that may be linked to pathologies that are similar to the various dysregulations and multisystemic manifestations in CF. Studies published from 2001 to 2022 were identified through the PubMed, Scopus, and Web of Science databases, and screening was performed following the PRISMA guidelines. The included studies were assessed using a quality checklist. From the 774 records identified, 28 studies met the inclusion criteria. Although no evidence has been found directly related to bone loss in CF individuals, some studies revealed a positive association between leptin levels and BMD, while others found an inverse association. Current evidence suggests that for circulating leptin levels to be a predictive biomarker of bone health, further research will be needed to reveal the direct and indirect mechanisms behind leptin and bone loss and to understand whether changes in leptin levels correlate with changes in BMD. Of note, studies with CF people would be of high importance to understand the role of leptin in CF-related bone disease.

## 1. Introduction

Leptin is secreted by mature adipocytes in white adipose tissue and its levels are directly proportional to the amount of white adipose tissue [1,2]. Its main function is the regulation of appetite and energy intake. Nevertheless, this adipokine has other effects in the body, namely, in bone homeostasis [1]. Of note, circulating leptin levels require tight tuning, as both hyper- and hypoleptinemia have been associated with a pro-inflammatory state, increasing susceptibility to infections, autoimmune diseases and inflammatory responses [3]. Regarding bone homeostasis, leptin promotes osteoblast cell proliferation and differentiation while hampering osteoclast formation, suggesting an important role in the pathophysiology of bone diseases [4,5]. Therefore, leptin seems to improve bone anabolism while decreasing bone catabolism. Osteopenia or low bone mass are usually due to dysregulation of bone metabolism, such as increased bone degradation and decreased bone formation [6].

Cystic fibrosis (CF) patients have higher levels of leptin [7] and are at a higher risk of developing osteopenia and osteoporosis through a multifactorial and yet not clearly understood mechanism [6,8,9]. Bone tissue dysregulation may be influenced by genetic defects in cystic fibrosis transmembrane conductance regulator (CFTR), pro-inflammatory status, among other factors [6]. Cystic fibrosis bone disease (CFBD) has devastating consequences for the quality of life and longevity of CF individuals [6]. A key component of minimizing the negative effects of low bone mass is early detection of bone alterations in CFBD. According to American Cystic Fibrosis Foundation and European Cystic Fibrosis Society guidelines, bone density scans are a routine assessment of bone mineralization that is crucial to providing useful information about body composition [8,10,11,12]. Measurement of bone mineral density (BMD) by dual-energy X-ray absorptiometry (DEXA or DXA) is considered the gold standard non-invasive method. It is recommended that a baseline assessment of BMD be performed before puberty, followed by periodic reassessments based on those results [12,13]. However, DEXA has some limitations, such as the incapability to detect alterations in bone microarchitecture, the exposure to X-rays, and its high cost. Moreover, only 66% of adults with CF undergo DEXA in the US [14]. This evidence highlights the risk of underdiagnosis of CFBD, as well as the need for better biomarkers to stratify patients and predict the risk of developing bone disease [15]. For example, it would be more advantageous to find a biomarker present in the blood, allowing BMD monitoring through routine venipuncture during the clinical appointment. CF individuals have higher levels of leptin in circulation and those levels correlate inversely with lung function [7]. Moreover, leptin has been associated with BMD and fracture risk [1,2,16,17,18,19]. However, it is still unknown whether circulating leptin levels are associated with low bone mineral density and fracture risk in cystic fibrosis. Therefore, it would be important to analyze the effect of leptin on CFBD and observe whether there is a correlation with BMD. This systematic review aims to summarize the current evidence in this area. In this context, two important points will be analyzed and discussed: (a) the association between leptin and BMD, and (b) the importance of leptin levels as a biomarker of bone health. In addition, a quality assessment checklist will be used to assess the reporting quality of the included studies.

## 2. Materials and Methods

### 2.1. Protocol, Registration and Search Strategy

**Figure 1:**
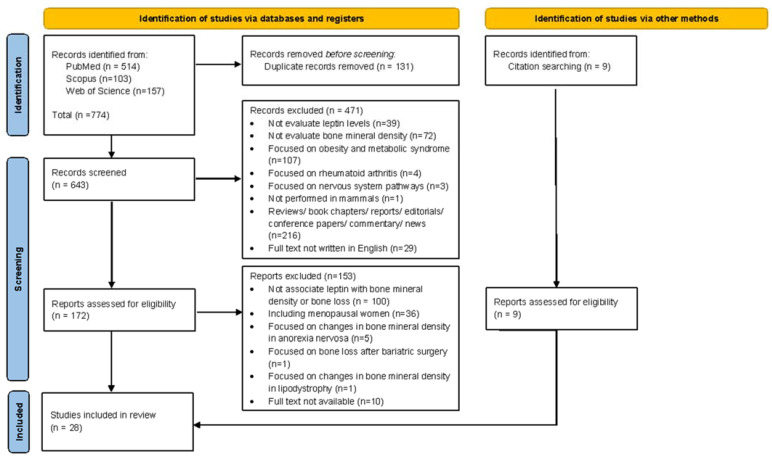
Flow diagram of the studies’ selection and data collection process.

Published studies were screened following the recommendations of the Preferred Reporting Items for Systematic Reviews and Meta-Analysis (PRISMA) Guidelines [20,21] (Tables S1 and S2). The protocol was developed according to the Preferred Reporting Items for Systematic Review and Meta-Analysis Protocols (PRISMA-P) guidelines [22] and registered in the International Prospective Register of Systematic Reviews (PROSPERO; registration number CRD42025631901). PubMed (https://pubmed.ncbi.nlm.nih.gov/; accessed on 20 October 2024), Scopus (https://www.scopus.com; accessed on 20 October 2024), and Web of Science (https://www.webofscience.com; accessed on 20 October 2024) databases were searched from 2001 to 20 October 2024, with the following keywords or medical subject heading (MeSH) terms: (leptin) AND (low bone mineral density AND bone loss). No publication date or language restrictions were applied at this stage. The reference lists of selected studies were manually reviewed to ensure that all relevant articles were identified and included. The procedure followed to select studies is presented in the PRISMA flow diagram (Figure 1).

### 2.2. Eligibility Criteria

The inclusion criteria were (1) studies concerning the influence of leptin on bone mass; and (2) studies regarding circulating leptin levels associated with low bone mineral density. The exclusion criteria were as follows: (i) review articles, letters, case reports, editorials, book chapters, or conference papers; (ii) studies not performed in mammals; (iii) studies that did not evaluate the leptin levels; (iv) studies that did not evaluate the bone mineral density; (v) studies focused on obesity and metabolic syndrome; (vi) studies focused on rheumatoid arthritis; (vii) studies focused on nervous system pathways; (viii) studies including postmenopausal women; (ix) studies focused on changes in BMD in anorexia nervosa; (x) studies focused on bone loss after bariatric surgery; (xi) articles where full text was not available; and (xii) studies not written in the English language. For further explanation on the exclusion criteria please see the discussion section.

### 2.3. Study Selection and Data Collection Process

The reference list of the selected studies was screened independently by two reviewers (DC and ACG) according to the eligibility criteria. In a first phase, titles and abstracts were carefully analyzed, and studies that failed to meet the inclusion criteria were excluded immediately from further analysis. In a second phase, the full texts of the remaining studies were evaluated to determine their inclusion or exclusion. The reference lists of full articles that met inclusion criteria were then compared, and any discrepancies were resolved through discussion and consensus. There were no disagreements that required consultation with a third reviewer. The following information was collected from each selected study: authors’ names; publication year; population studied; study design; number of individuals/animals enrolled in the study; methodology related to leptin and BMD assessment; disease assessed in the study and main findings associated with leptin and BMD.

### 2.4. Quality Assessment

The quality assessment of the included studies was determined according to a quality checklist developed specifically for basic science studies by Cosme et al. [23] (Table 1 and Table 2). The authors (DC and ACG) applied the checklist with 14 criteria independently. No disagreements were found. Each criterion of the checklist was scored as follows: information not available in the paper (0 points); limited information provided (1 point); and complete information regarding that aspect (2 points). For each study, the scores of all criteria were added and divided by the maximum score (28 points) to obtain the overall quality score of the articles.

## 3. Results

### 3.1. Literature Search and Study Selection

The initial electronic database search yielded 774 records (514 records in PubMed, 103 in Scopus, and 157 in ISI Web of Science), of which 131 were excluded for being duplicates (Figure 1). Based on titles and abstracts, 643 records were screened, and 471 studies were excluded. The remaining 172 studies were eligible for full text analysis (Figure 1). From these, 62 were excluded for not evaluating leptin levels associated with low BMD, 38 for not evaluating the influence of leptin on bone mass, 36 including postmenopausal women, 5 focused on changes in BMD in anorexia nervosa patients, 1 focused on bone loss after bariatric surgery, 1 focused on changes in BMD in lipodystrophy, and 10 for not having available full text (Figure 1). The manual search of relevant citations identified 9 additional studies. Overall, a total of 28 [24,25,26,27,28,29,30,31,32,33,34,35,36,37,38,39,40,41,42,43,44,45,46,47,48,49,50,51] studies were selected for inclusion in this systematic review.

### 3.2. Description of the Studies

The relevant data from each study are systematized in two tables, one including studies performed in humans (Table 1) and another comprising the findings from animal studies (Table 2). Regarding the studies included, leptin levels were quantified essentially by enzyme-linked immunoassay (ELISA) and radioimmunoassay (RIA). All included studies evaluate the BMD of individuals and the majority of the assessment was performed using dual energy X-ray absorptiometry (DEXA).

The included articles can be essentially divided into two main groups, observational and experimental study designs. Observational studies were mostly cross-sectional, case/control studies, also known as “retrospective studies”, which assessed associations between disease and exposure at one time point; and prospective cohort studies, a type of longitudinal study in which individuals are followed over a period of time [52,53]. The experimental study group randomly assigned the animal models in which the test group received the experimental intervention and the control group received a placebo or no treatment [52,53].

In this systematic review, the quality assessment performed for all included studies (Table 3, Table 4 and Table 5) revealed a mean quality score of 89% ± 0.29. The scores ranged from 75% [26,27] to 100% [39,48] (Table 3, Table 4 and Table 5 and Table S3).

Considering the 14 defined criteria, the criteria with lower scores are definition of the endpoints of the study within the “Purpose and hypothesis” section; evaluation by independent observers; blinding; evidence of independent repetitions” allocated to “Data collection” section and disclose conflicts of interest and declaring funding sources within “Analyzing data and manuscript drafting” section.

Based on the characterization of the included studies (Table 1 and Table 2), we can highlight that none of the studies were related to CF or studied CF people. Nevertheless, some considered diseases related to the multisystemic manifestations of CF, such as lung disease [36,40], liver dysfunction [24,37], and diabetes [45]. These studies present evidence linking leptin and BMD between patients and healthy individuals in population samples. In line with the aim of this systematic review, scientific evidence was focused on the association of leptin with low BMD.

### 3.3. Association Between Leptin Levels and Bone Mineral Density: Evidence from Human Studies

#### 3.3.1. Studies with Negative Association Between Leptin Levels and Bone Mass

The first study included in this systematic review to address was a case/control study of 58 adult patients with chronic liver disease and 54 healthy controls [24]. It demonstrated that leptin levels were negatively correlated with BMD at the lumbar spine and femoral neck in patients with chronic liver disease [24]. These results were later confirmed in chronic liver disease patients without renal disease [37]. In patients with chronic obstructive pulmonary disease, BMD was also negatively related with leptin levels [40]. Anagnostis et al. found that leptin levels were negatively associated with BMD at the femoral neck and total hip in hemophiliac men in a cross-sectional study [41]. More recently, Normand et al. carried out a cross-sectional pilot study in adolescents with idiopathic scoliosis and observed that leptin levels were higher in the patient group, but no association with BMD was observed [46]. In contrast, in the healthy individuals, it was observed that leptin levels were inversely correlated with BMD [46]. A cross-sectional study with patients with Duchenne muscular dystrophy demonstrated that these patients had higher levels of leptin than the healthy individuals, but had reduced BMD and reduced bone turnover markers [34]. Additionally, some observational studies conducted in healthy individuals, both young [32] and adult [25,29] men, revealed an inverse association between leptin and BMD.

#### 3.3.2. Studies with Positive Association Between Leptin Levels and Bone Mass

Despite the previous results with negative associations, an observational study of 363 healthy adult men found a positive correlation between BMD and leptin levels [27]. However, the leptin levels of men with normal and low BMD did not differ significantly. Along these lines, a population analysis that included 117 healthy infants quantified leptin through umbilical cord blood and observed a positive correlation between leptin and whole-body mineral content, bone area and estimated volumetric BMD [28]. Through a cross-sectional and longitudinal study, Crabbe and colleagues demonstrated that there was no significant association between leptin and baseline BMD in the hip and forearm of elderly men [31]. However, this study reports that, prospectively, forearm BMD loss was positively associated with leptin levels, suggesting that age may be an interplay between leptin levels and bone loss. Female adolescents with idiopathic scoliosis with low BMD showed reduced leptin levels compared to healthy female controls [33]. Moreover, this work reports that BMD in the lumbar spine and femoral neck is positively associated with leptin levels [33]. In a cross-sectional study, leptin levels in male patients with chronic obstructive pulmonary disease (COPD) directly correlated with BMD [36]. Furthermore, the COPD patients with osteoporosis had lower leptin levels compared to those without osteoporosis. Similar conclusions were obtained in a single-center prospective study with inflammatory bowel disease patients [38]. More recently, Ho-Pham and colleagues observed that higher leptin levels were positively associated with BMD at the lumbar spine and femoral in both women and men [44]. These authors also reported that in healthy males, leptin levels were also correlated with whole-body BMD. Additionally, Brown et al. showed through univariate analysis that higher leptin levels were associated with increased BMD in patients infected with human immunodeficiency virus [42]. In a placebo-controlled study, long-term leptin administration in lean lypoleptinemic women led to an increase in lumbar spine BMD and bone mineral content [39].

#### 3.3.3. Studies with No Association Between Leptin Levels and Bone Mass

Another group of studies found no association between leptin levels and BMD [26,30,35,43,45]. A study encompassing 105 healthy female adolescents concluded that leptin levels were not associated with total body BMD and bone mineral content [26]. Additionally, a retrospective study that included 20 survivors of childhood leukemia and lymphoma and 20 healthy children revealed that leptin levels were not correlated with BMD and osteocalcin, a marker of bone formation [30]. On the other hand, a prospective study conducted in 57 preterm newborns measured the cord blood serum leptin levels and found that cord blood serum leptin were significantly lower when compared to full-term newborns, but no association was found with lumbar spine BMD during the first two years [43]. Similar findings were obtained in a study of mothers with gestational diabetes or type 2 diabetes and normoglycemia, in which cord blood leptin levels of their infants were not associated with infant BMD [45]. Peng et al. in an observational study including 232 healthy men, also found no association between leptin levels and BMD [35].

In the quality assessment of the included studies performed in humans, six articles obtained a score above 90% [28,36,38,40,45,46] and only one article achieved the best score of 100% [39] (Table 3, Table 4, Table 5 and Table S3). In this set of publications, some articles have scores below 90% [24,25,26,27,29,30,31,32,33,34,35,37,41,42,43,44], which can be justified by the lack of information about the purpose and hypothesis and study design.

### 3.4. Impact of Leptin Administration on Bone Mineral Density: Evidence from Animal Studies

Although most published studies are observational and focus on the association of leptin with BMD in humans, some research has been performed in experimental animal models. Martin and colleagues, in 2015, observed that daily administration of 0.35 mg leptin per kg during 14 days prevented a progressive decrease in BMD in tail-suspended female Wistar rats [48]. Two years later, the same research team observed that a lower dose (50 µg/kg·day) of leptin administration was able to prevent the suspension-induced bone loss [49]. Given these results, it can be concluded that the effect of leptin on bone metabolism was dose dependent. In turn, Stunes et al. demonstrate that treatment with 100 µg/day of leptin resulted in a significant reduction in BMD in female Fisher rats [51]. According to Motyl et al. in diabetic rodent models, leptin levels were reduced and leptin replacement was not effective in preventing bone loss [50]. A study performed on rodents treated with clenbuterol, a type 2 adrenergic agonist, showed a decrease in leptin levels and BMD, and an increase in C-terminal collagen crosslink, a bone-resorption marker [47].

These experimental studies scored highly on the quality assessment. Two articles scored 93% [47,51], two other articles scored 96% [49,50], and one article scored 100% [48] (Table 3, Table 4, Table 5 and Table S3).

## 4. Discussion

Leptin has been linked to direct or indirect involvement in bone metabolism [1]. However, despite the scientific evidence published in recent years, it is still unknown whether leptin levels associate with BMD. To help answer this question, we analyzed and integrated current evidence on the relationship between circulating leptin levels and BMD. Even though no study directly studied CF individuals or focused on CFBD, several studies focused on pathologies that are similar to the various dysregulations and multisystemic manifestations in CF. Furthermore, we also considered studies that included healthy individuals. Additionally, a qualitative assessment of the included studies was also performed.

To minimize the possibility of bias in this systematic review, we excluded studies related to various manifestations/diseases unrelated to CF manifestations, such as obesity and metabolic syndrome, rheumatoid arthritis, nervous system pathways, postmenopausal osteoporosis, anorexia nervosa, bariatric surgery, and lipodystrophy. The results of these studies may give misleading conclusions regarding the association between leptin and BMD and are difficult to translate to CFBD. According to previous systematic reviews and meta-analyses performed in healthy individuals, leptin levels were positively associated with BMD, but this association was dependent on the menopausal status of women [16,17]. In published articles, leptin appears to be positively correlated with BMD, especially in postmenopausal women [16,17,18]. Studies including postmenopausal women have shown that high levels of leptin are associated with higher BMD levels and a lower risk of bone fractures [17,18]. Therefore, with the intention of minimizing bias in this review, we did not include studies involving menopausal and postmenopausal women. Our decision was based on data from the 2023 Cystic Fibrosis Foundation Registry, as the average age of death for individuals with CF was 36.9 years [9], indicating that most female CF individuals are at a pre-menopausal status. Nevertheless, the average survival age for CF individuals is increasing with improved CFTR modulator therapy, and for those born between 2019 and 2023, the prediction is 61 years [9]. Given that the average survival age in CF individuals is increasing, we considered studies that included middle-aged and elderly men to observe the association between leptin and BMD. Additionally, the impact of estrogen deprivation in CF women after menopause on bone health and CFBD should be addressed in future studies.

### 4.1. Evidence from Observational Studies

In this systematic review, evidence from human studies was analyzed separately from that performed in animal models to facilitate comparison of the main results. The studies included here approach the association of leptin levels with BMD in several pathologies: chronic obstructive pulmonary disease [36,40], chronic liver disease [24,37], chronic renal disease [37], type 2 diabetes mellitus [45], inflammatory bowel disease (IBD) [38], idiopathic scoliosis [33,46], Duchenne muscular dystrophy [34], and hypoleptinemia [39]. Additionally, the association between leptin and BMD has also been evaluated in healthy individuals: men, both young [32], adults [25,27,29,44], and elderly [31]; women, both young [26] and adults [44]; and infants [28,30,43].

Observational studies have shown that the association between leptin and BMD is still controversial. Despite the inconsistency in the results, this review reveals that considering individuals with associated pathology, the available studies reported a positive association between leptin levels and BMD. Evidence shows that a deterioration in lung function is correlated with a reduction in BMD [36], which is similar to CFBD because lung function and its decay have been correlated with low BMD [54,55,56,57,58,59,60]. Leptin levels were lower in men with obstructive pulmonary disease and osteoporosis than in those without osteoporosis [36]. To our best knowledge, the levels of leptin have not been compared in CF patients regarding BMD status. We hypothesize that future studies focusing on this relationship should be carried out. Along these lines, patients with IBD and osteoporosis had lower leptin levels compared to normal BMD patients [38]. Therefore, reduced leptin levels may decrease bone formation and increase bone resorption. Other studies have associated reduced BMD with advanced age in men [31]. CFBD is mostly diagnosed in adults, but bone defects may appear early in life [61,62] and progress with age [63]. Of note, in healthy individuals (men, women, and infants), circulating leptin levels are also positively associated with BMD [27,28,44].

Some studies have reported higher leptin levels in patient groups compared to control groups [34,37,46]. Circulating leptin levels are also increased in CF patients compared to healthy individuals [15]. Yet, the impact of these higher levels on bone health remains unexplored. On the other hand, one of the studies included in this systematic review reported a negative correlation between leptin and BMD in patients with chronic liver disease [37]. Other studies did not find a consensual association between leptin levels and BMD in adolescents with idiopathic scoliosis [33,46] and Duchenne muscular dystrophy [34]. While patients with Duchenne muscular dystrophy had a reduction in BMD and markers of bone formation and bone resorption [34], adolescents with idiopathic scoliosis and low BMD presented two scenarios: BMD and leptin were positively correlated in one of the studies [33], with no correlation in the second [46]. The remaining literature found no significant association between leptin and BMD. These studies highlight the importance of addressing leptin levels in the context of each pathology and always having in mind the population studied. Moreover, it is possible that the association between leptin levels and BMD is not direct and depends on other factors. This concept is particularly relevant in CFBD due to its multifactorial etiology [6].

### 4.2. Evidence from Experimental Studies

Regarding experimental studies using animal models, the consistency of published data follows the same line. The effect of leptin on BMD is dose- and time-dependent [48,49,51]. These observations raise the question of whether leptin effects on the bone are similar to parathyroid hormone (PTH), as continuous exposure to high levels of PTH leads to bone resorption but intermittent exposure to PTH promotes bone anabolism [64]. Nevertheless, type 1 diabetic rodents had reduced circulating leptin levels, and leptin replacement did not prevent bone loss [50]. Yet, the lack of effect of leptin replacement may be obscured by the endocrine dysfunction. This is particularly important in CFBD, as CF-related diabetes may impact bone loss [65]. Along these lines, hypoleptinemic women receiving long-term leptin administration had an increase in BMD at the lumbar spine [39]. Overall, the lack of consistency in the results and the reduced number of studies prompt that more research is needed.

### 4.3. Limitations

To date, as far as the authors know, no systematic review has been performed on the association between leptin and BMD with a focus on CFBD. This systematic review was performed according to the PRISMA guidelines [20], and the results of our analysis are reliable and useful for the next steps of sustained research in this field. Nevertheless, this systematic review has some limitations that should be considered. Firstly, despite the best efforts to optimize search strategies, 9 articles did not appear in electronic search. Possibly, this may be due to difficulties in keyword-based indexing services that are related to adequate keyword selection, which should be as closely as possible in line with medical subject headings and subheadings. Secondly, as a result of the heterogeneity found in the study design used in the included articles, it is difficult to generalize and compare the main findings in a suitable systematic review. Furthermore, several studies performed a cross-sectional analysis with short follow-up periods. In addition, there are few quantitative data available, which limits the potential for meta-analysis.

## 5. Conclusions

Overall, the available data do not clearly associate leptin levels with BMD due to the discrepancy of the results found. Some studies point to a positive association between leptin levels and BMD, while others reveal an inverse association. Several studies associate leptin and BMD, but none directly aimed to address the direct relationship between leptin levels and bone metabolism. While we found no evidence directly related to bone loss in CF patients, several studies included pathologies similar to CF-related disorders as well as multifactorial complications of CF. Yet, further studies using cohorts of CF patients need to be performed to dissect whether or not leptin levels correlate with alterations in bone mineral density and whether this association is modified by treatment with CFTR modulators.

The applicability of leptin as a biomarker for bone health has not been addressed. It will be important to determine whether changes in leptin levels correlate with changes in bone mineral density and architecture. This would be particularly important for CF-related bone disease due to the gap in surveillance of bone health and diagnosis of bone disease.

## Figures and Tables

**Table 1:** Characterization of the included studies performed in humans.

Reference	Population	Study Design	Individuals, *n*	Disease Assessed	Methods	Main Findings Associated with Leptin and BMD
Ormarsdóttir et al., 2001 [24]	Sweden	Case/Control	58 adult patients; 54 healthy controls	Chronic liver disease	BMD was measured by DEXA; serum leptin levels by RIA	Leptin correlated negatively with BMD at lumbar spine and the femoral neck in patients with advanced chronic liver disease.
Sato et al., 2001 [25]	Japan	Observational	221 healthy adult men	None	BMD was measured by single photon absorptiometry; Serum leptin levels by RIA	Leptin was inversely associated with BMD of the calcaneus after adjustment for body weight.
Huang et al., 2004 [26]	China	Observational	105 female adolescents	None	BMD and BMC were measured by DEXA; serum leptin levels by RIA	Leptin levels were not related to the total body BMD and BMC.
Papadopoulou et al., 2004 [27]	Greece	Observational	363 healthy adult men	None	BMD was measured by DEXA; serum leptin levels by two-site immunoradiometric assay	BMD and leptin levels were positively correlated. Leptin levels were not significantly different between men with normal BMD and low BMD.
Javaid et al., 2005 [28]	UK	Population-based	117 healthy infants	None	BMD was measured by DEXA; serum leptin levels by RIA	Umbilical cord leptin concentration was positively correlated with whole body bone mineral content, bone area, and estimated volumetric BMD.
Oh et al., 2005 [29]	Korea	Cross-sectional	80 healthy men	None	BMD was measured by DEXA; serum leptin levels by RIA	A significant negative correlation was observed between log-transformed leptin levels and lumbar spine BMD after adjusting for age and body mass index.
Yaris et al., 2005 [30]	Turkey	Retrospective	20 pediatric patients; 20 healthy children	Acute lymphoblastic leukemia or non-Hodgkin lymphoma	BMD was measured by DEXA; serum leptin levels by ELISA	Leptin levels were not correlated with BMD and markers of bone metabolism (osteocalcin) by multivariate analysis. In contrast, in a simple correlation analysis, leptin and BMD had a significant association.
Crabbe et al., 2006 [31]	Belgium	Cross-sectional and longitudinal	270 elderly men	None	BMD was measured by DEXA; serum leptin levels by RIA	No significant association between leptin and baseline BMD at the hip and forearm. Prospectively, BMD loss was not associated with serum leptin at the hip. In contrast, at the forearm BMD loss was positively associated with leptin.
Lorentzon et al., 2006 [32]	Sweden	Population-based	1068 healthy young men	None	BMD was measured by DEXA; serum leptin levels by ELISA	Leptin was a negative independent predictor of areal BMD and cortical bone size of the radius and tibia.
Qiu et al., 2007 [33]	China	Cross-sectional	120 female patients; 80 healthy female controls	Adolescent idiopathic scoliosis	BMD was measured by DEXA; serum leptin levels by ELISA	Reduced leptin levels were associated with lower bone mass in patients with adolescent idiopathic scoliosis. Positive association between leptin and BMD at the lumbar spine and femoral neck.
Söderpalm et al., 2007 [34]	Sweden	Cross-sectional	24 male patients; 24 healthy male controls	Duchenne muscular dystrophy	BMD was measured by DEXA; serum leptin levels by RIA	Leptin levels were significantly higher in the Duchenne muscular dystrophy patient group than in the control group. The patient group had reduced BMD and reduced bone turnover markers (bone formation: BALP, PINP, osteocalcin; bone resorption: CTX and TRACP5b).
Peng et al., 2008 [35]	China	Observational	232 healthy men	None	BMD was measured by DEXA; serum leptin levels by ELISA	Leptin levels were not significantly correlated with BMD.
Vondracek et al., 2009 [36]	USA	Cross-sectional	23 male patients	Chronic obstructive pulmonary disease	BMD was measured by DEXA; serum leptin levels by ELISA	Leptin levels were significantly lower in men with osteoporosis. Leptin was positively correlated with BMD.
Ghonemy et al., 2011 [37]	Egypt	Case/Control	20 patients with end-stage renal disease (ESRD); 20 patients with chronic liver disease (CLD); 20 patients with ESRD + CLD; 20 healthy controls	End-stage renal disease and chronic liver disease	BMD was measured by DEXA; serum leptin levels by solid phase Enzyme Amplified Sensitivity Immunoassay	Leptin levels increased in all patient groups compared to the control group. Leptin was negatively correlated with BMD in chronic liver disease patients without renal disease.
Koutroubakis et al., 2011 [38]	Greece	Prospective	118 adults	Inflammatory bowel disease	BMD was measured by DEXA; serum leptin levels by RIA	Serum leptin levels were significantly lower in IBD patients with osteoporosis compared with osteopenia and normal BMD patients. Through univariate analysis, leptin levels were positively correlated with BMD at the femoral neck and lumbar spine. In a multivariate analysis, no independent correlation was observed between leptin and BMD.
Sienkiewicz et al., 2011 [39]	USA	Placebo-controlled	20 adult women	Hypoleptinemia and hypothalamic amenorrhea	BMD was measured by DEXA; serum leptin levels by ELISA	Long-term leptin replacement with recombinant human methionyl leptin increased lumbar spine BMD and BMC of lean young women with hypoleptinemia, as well as altered the bone remodeling environment to promote bone formation.
Fountoulis et al., 2012 [40]	Greece	Cross-sectional	46 male patients	Chronic obstructive pulmonary disease	BMD was measured by DEXA; serum leptin levels by RIA	Whole body T-score was negatively related to leptin and chronic obstructive pulmonary disease stage.
Anagnostis et al., 2013 [41]	Greece	Cross-sectional	81 male patients	Haemophilia	BMD was measured by DEXA; serum leptin levels by ELISA	Leptin levels were negatively associated with BMD at the femoral neck and total hip in hemophiliac men.
Brown et al., 2013 [42]	USA and Puerto Rico	Cross-sectional	331 patients	Human immunodeficiency virus infection	BMD was measured by DEXA; serum leptin levels by ELISA	Higher leptin levels were associated with increased Z-score BMD by univariate analysis. In multivariable analysis, associations with leptin levels were no longer statistically significant.
Veselá et al., 2016 [43]	Czech Republic	Prospective	57 preterm newborns	None	BMD was measured by DEXA; serum leptin levels by ELISA	Leptin levels were significantly lower in the cord blood of preterm newborns than in term-delivered newborns. Lower leptin levels were not associated with lumbar spine BMD in cord blood and serum of preterm infants during the first 2 years of life.
Ho-Pham et al., 2017 [44]	Vietnam	Cross-sectional	611 healthy adults	None	BMD was measured by DEXA; serum leptin levels by ELISA	In women, higher leptin levels were positively associated with lumbar spine and femoral neck BMD, but not with whole body BMD. At all three BMD sites, leptin levels were also correlated with BMD in men.
Krishnan et al., 2022 [45]	USA	Prospective	64 patients; 94 mothers with normoglycemia as controls	Gestational diabetes or type 2 diabetes	BMD was measured by DEXA; serum leptin levels by ELISA	Cord blood leptin levels were not associated with infant bone mass.
Normand et al., 2022 [46]	Canada	Cross-sectional pilot	21 female patients; 19 age-matched healthy controls	Adolescent idiopathic scoliosis	BMD was measured by DEXA; serum leptin levels by multiplex assay	Leptin levels were higher in patients with adolescent idiopathic scoliosis compared to controls. No association between leptin levels and BMD was observed in the patient group. In contrast, leptin levels inversely correlated with BMD in the control group.

BMD, bone mineral density; BALP, bone-specific alkaline phosphatase; DEXA, dual energy X-ray absorptiometry; ELISA, enzyme-linked immunosorbent assay; PINP, serum type I procollagen intact amino-terminal propeptide; RIA, radioimmunoassay; TRACP5b, acid phosphatase isoform 5b.

**Table 2:** Characterization of the included studies performed in animal models.

Reference	Study Design	Animals, *n*	Disease Assessed	Methods	Main Findings Associated with Leptin and BMD
Bonnet et al., 2005 [47]	Case-control	39 female Wistar rats, divided in 3 groups (salbutamol, clenbuterol and control)	None	BMD and BMC were measured by DEXA; serum leptin levels by ELISA	Animals treated with clenbuterol, a selective β2 adrenergic agonist, had lower leptin plasma levels and lower bone density. β2 adrenergic agonists increased C-terminal collagen crosslinks, a bone resorption marker, without changing osteocalcin levels, a bone formation marker.
Martin et al., 2005 [48]	Interventional	130 female Wistar rats, divided in 13 groups (tail-suspended or non-suspended and treated with leptin or vehicle)	None	BMD was measured by DEXA; serum leptin levels by ELISA	A two-week administration of leptin prevented a progressive decrease in tibial metaphysis BMD in tail-suspended rats.
Martin et al., 2007 [49]	Interventional	70 female Wistar rats, divided in 7 groups (tail-suspended or non-suspended and treated with leptin or vehicle)	None	BMD was measured by DEXA; serum leptin levels by ELISA	Low-dose leptin administration for 14 days prevented the trabecular and cortical bone loss in tail-suspended rats. In contrast, high-dose leptin administration reduced bone mass and inhibited femoral bone growth in both tail-suspended and non-suspended groups.
Motyl et al., 2009 [50]	Case-control	40 BALB/c mice, divided in 4 groups (control + vehicle, control + leptin, diabetic + vehicle and diabetic + leptin)	Type 1 diabetes	BMD and BMC were measured by micro computed tomography; serum leptin levels by enzyme immunometric assay	Serum leptin levels were reduced in mice with type 1 diabetes. Leptin treatment did not prevent bone loss in diabetic rodent models.
Stunes et al., 2012 [51]	Case-control	45 female Fisher rats, divided in 3 groups (low-dose leptin, high-dose leptin and control)	None	BMD and BMC were measured by DEXA; serum leptin levels by RIA	Low-dose leptin treatment resulted in a significant reduction in whole-body BMD and reduced bone strength.

BMD, bone mineral density; BMC, bone mineral content; DEXA, dual energy X-ray absorptiometry; ELISA, enzyme-linked immunosorbent assay; RIA, radioimmunoassay.

**Table 3:** Assessment of the reporting inclusiveness of the included studies; scored as 0 points (information not available in the paper); 1 point (limited information provided); 2 points (complete information regarding that aspect).

Reporting Assessment		Ormarsdóttir et al. [24]	Sato et al. [25]	Huang et al. [26]	Papadopoulou et al. [27]	Javaid et al. [28]	Oh et al. [29]	Yaris et al. [30]	Crabbe et al. [31]	Lorentzon et al. [32]	Qiu et al. [33]	Söderpalm et al. [34]
Criteria												
Problem definition	1. Scientific background and explanation of rationale	2	2	1	2	2	2	2	2	2	2	2
Purpose and hypothesis	2. Definition of the specific objectives or hypotheses	2	2	2	2	2	2	2	2	2	2	2
	3. Definition of the endpoints to study	1	1	1	1	2	2	2	1	2	1	1
Study design	4. Accurate description of the laboratory methodologies (easy to understand and described in enough detail to allow replication), definition of the test compounds, experimental conditions and other important information; use of validated methods	2	2	2	1	2	1	1	1	2	2	2
	5. Ethical review permissions, when applicable	2	2	2	2	2	2	2	2	0	2	2
	6. Description of the statistical methods, when adequate	2	2	2	2	2	2	2	2	2	2	2
Data collection	7. Obtain valid data and ensure that it is reliable	2	2	2	2	2	2	2	2	2	2	2
	8. Evaluation by independent observers; blinding; evidence of independent repetitions	1	0	1	1	1	0	0	1	1	0	1
Analysing data and manuscript drafting	9. Cite relevant scientific papers when presenting evidence	2	2	2	2	2	2	2	2	2	2	2
	10. Accessible and transparent presentation of data throughout the paper (including the appropriate measures of precision/variance)	2	2	2	2	2	2	2	2	2	2	2
	11. Critical discussion of the results; comparison with relevant research on the field	2	2	2	2	2	2	2	2	2	2	2
	12. Draw consistent conclusions based on the evidence presented in the paper	2	2	2	1	2	2	2	2	2	2	2
	13. State the contribution to cumulative scientific knowledge and the practical implications of the findings	1	2	1	1	2	2	1	2	2	2	2
	14. Disclose conflicts of interest and declaring funding sources	0	0	1	0	1	1	0	1	1	2	0
Overall score		1.64	1.64	1.64	1.50	1.86	1.71	1.57	1.71	1.71	1.79	1.71
Standard deviation		0.61	0.72	0.48	0.63	0.35	0.59	0.73	0.45	0.59	0.56	0.59
Overall score/Maximum score		82%	82%	82%	75%	93%	86%	79%	86%	86%	89%	86%

**Table 4:** Assessment of the reporting inclusiveness of the included studies; scored as 0 points (information not available in the paper); 1 point (limited information provided); 2 points (complete information regarding that aspect). (*Cont*.).

Reporting Assessment		Peng et al. [35]	Vondracek et al. [36]	Ghonemy et al. [37]	Koutroubakis et al. [38]	Sienkiewicz et al. [39]	Fountoulis et al. [40]	Anagnostis et al. [41]	Brown et al. [42]	Veselá et al. [43]	Ho-Pham et al. [44]
Criteria											
Problem definition	1. Scientific background and explanation of rationale	1	2	2	2	2	2	2	2	2	2
Purpose and hypothesis	2. Definition of the specific objectives or hypotheses	1	2	1	2	2	2	2	1	2	2
	3. Definition of the endpoints to study	2	2	1	1	2	2	2	1	2	1
Study design	4. Accurate description of the laboratory methodologies (easy to understand and described in enough detail to allow replication), definition of the test compounds, experimental conditions and other important information; use of validated methods	2	2	2	2	2	2	2	1	2	2
	5. Ethical review permissions, when applicable	2	2	2	2	2	2	2	2	2	2
	6. Description of the statistical methods, when adequate	2	2	2	2	2	2	2	2	2	2
Data collection	7. Obtain valid data and ensure that it is reliable	2	2	2	2	2	2	2	2	2	2
	8. Evaluation by independent observers; blinding; evidence of independent repetitions	1	1	2	2	2	2	1	0	1	1
Analysing data and manuscript drafting	9. Cite relevant scientific papers when presenting evidence	2	2	2	2	2	2	2	2	2	2
	10. Accessible and transparent presentation of data throughout the paper (including the appropriate measures of precision/variance)	2	2	2	2	2	2	2	2	1	2
	11. Critical discussion of the results; comparison with relevant research on the field	2	2	2	2	2	2	2	2	2	2
	12. Draw consistent conclusions based on the evidence presented in the paper	2	2	2	2	2	2	2	2	2	1
	13. State the contribution to cumulative scientific knowledge and the practical implications of the findings	2	2	1	2	2	2	1	2	2	1
	14. Disclose conflicts of interest and declaring funding sources	1	2	1	2	2	0	0	2	1	2
Overall score		1.71	1.93	1.71	1.93	2.00	1.86	1.71	1.64	1.79	1.71
Standard deviation		0.45	0.26	0.45	0.26	0.00	0.52	0.59	0.61	0.41	0.45
Overall score/Maximum score		86%	96%	86%	96%	100%	93%	86%	82%	89%	86%

**Table 5:** Assessment of the reporting inclusiveness of the included studies; scored as 0 points (information not available in the paper); 1 point (limited information provided); 2 points (complete information regarding that aspect). (*Cont*.).

Reporting Assessment		Krishnan et al. [45]	Normand et al. [46]	Bonnet et al. [47]	Martin et al. [48]	Martin et al. [49]	Motyl et al. [50]	Stunes et al. [51]	Average Score
Criteria									
Problem definition	1. Scientific background and explanation of rationale	2	2	2	2	2	2	2	1.91
Purpose and hypothesis	2. Definition of the specific objectives or hypotheses	1	2	2	2	2	2	2	1.83
	3. Definition of the endpoints to study	2	2	2	2	2	2	1	1.52
Study design	4. Accurate description of the laboratory methodologies (easy to understand and described in enough detail to allow replication), definition of the test compounds, experimental conditions and other important information; use of validated methods	2	2	2	2	2	2	2	1.78
	5. Ethical review permissions, when applicable	2	2	2	2	2	2	2	1.91
	6. Description of the statistical methods, when adequate	2	2	2	2	2	2	2	2.00
Data collection	7. Obtain valid data and ensure that it is reliable	2	2	2	2	2	2	2	2.00
	8. Evaluation by independent observers; blinding; evidence of independent repetitions	2	2	2	2	2	2	2	1.04
Analysing data and manuscript drafting	9. Cite relevant scientific papers when presenting evidence	2	2	2	2	2	2	2	2.00
	10. Accessible and transparent presentation of data throughout the paper (including the appropriate measures of precision/variance)	2	2	2	2	2	2	2	1.96
	11. Critical discussion of the results; comparison with relevant research on the field	2	2	2	2	2	2	2	2.00
	12. Draw consistent conclusions based on the evidence presented in the paper	2	1	2	2	2	2	2	1.87
	13. State the contribution to cumulative scientific knowledge and the practical implications of the findings	2	1	2	2	2	2	1	1.65
	14. Disclose conflicts of interest and declaring funding sources	2	2	0	2	1	1	2	1.04
Overall score		1.93	1.86	1.86	2.00	1.93	1.93	1.86	1.75
Standard deviation		0.26	0.35	0.52	0.00	0.26	0.26	0.35	0.32
Overall score/Maximum score		96%	93%	93%	100%	96%	96%	93%	88%

## Data Availability

Not applicable.

## Supplementary Materials

The following supporting information can be downloaded at: https://www.mdpi.com/article/10.3390/ijms26052066/s1.

## Abbreviations

The following abbreviations are used in this manuscript:

BALP	Bone-specific alkaline phosphatase
BMD	Bone mineral density
CF	Cystic fibrosis
CFBD	Cystic fibrosis bone disease
CFTR	Cystic fibrosis transmembrane conductance regulator
COPD	Chronic obstructive pulmonary disease
DEXA	Dual-energy X-ray absorptiometry
ELISA	Enzyme-linked immunoassay
MeSH	Medical subject heading
PINP	Serum type I procollagen intact amino-terminal propeptide
PRISMA	Preferred reporting items for systematic reviews and meta-analysis
PRISMA-P	Preferred reporting items for systematic Review and Meta-Analysis Protocols
PROSPERO	Prospective register of systematic reviews
PTH	Parathyroid hormone
RIA	Radioimmunoassay
TRACP5b	Acid phosphatase isoform 5b
