# Supplementary material for: Leptin Levels and Bone Mineral Density: A Friend or a Foe for Bone Loss? A Systematic Review of the Association Between Leptin Levels and Low Bone Mineral Density
Source: Int J Mol Sci. 2025 Feb 27;26(5):2066. doi: 10.3390/ijms26052066 (PMC11901020; doi:10.3390/ijms26052066)
Supplement: Supplementary file 1 [file ijms-26-02066-s001.zip › ijms-3465235 supplementary.pdf]

Table S1. PRISMA 2020 main checklist.

Topic	No.	Item	Location Where Item Is Reported
**TITLE**			
**Title**	1	Identify the report as a systematic review.	Title section, Line 2–4
**ABSTRACT**			
**Abstract**	2	See the PRISMA 2020 for Abstracts checklist.	
**INTRODUCTION**			
**Rationale**	3	Describe the rationale for the review in the context of existing knowledge.	Line 33–70
**Objectives**	4	Provide an explicit statement of the objective(s) or question(s) the review addresses.	Line 70–74
**METHODS**			
**Eligibility criteria**	5	Specify the inclusion and exclusion criteria for the review and how studies were grouped for the syntheses.	Line 92–102
**Information sources**	6	Specify all databases, registers, websites, organizations, reference lists and other sources searched or consulted to identify studies. Specify the date when each source was last searched or consulted.	Line 78–90 and Figure 1
**Search strategy**	7	Present the full search strategies for all databases, registers and websites, including any filters and limits used.	Line 83–87 and Figure 1
**Selection process**	8	Specify the methods used to decide whether a study met the inclusion criteria of the review, including how many reviewers screened each record and each report retrieved, whether they worked independently, and if applicable, details of automation tools used in the process.	Line 104–115
**Data collection process**	9	Specify the methods used to collect data from reports, including how many reviewers collected data from each report, whether they worked independently, any processes for obtaining or confirming data from study investigators, and if applicable, details of automation tools used in the process.	Line 104–115

**Data items**	10a	List and define all outcomes for which data were sought. Specify whether all results that were compatible with each outcome domain in each study were sought (e.g., for all measures, time points, analyses), and if not, the methods used to decide which results to collect.	Line 111–115 and Tables 1 and 2
	10b	List and define all other variables for which data were sought (e.g., participant and intervention characteristics, funding sources). Describe any assumptions made about any missing or unclear information.	Tables 1 and 2
**Study risk of bias assessment**	11	Specify the methods used to assess risk of bias in the included studies, including details of the tool(s) used, how many reviewers assessed each study and whether they worked independently, and if applicable, details of automation tools used in the process.	Line 117–124 and Tables 3-5
**Effect measures**	12	Specify for each outcome the effect measure(s) (e.g., risk ratio, mean difference) used in the synthesis or presentation of results.	Tables 3-5
**Synthesis methods**	13a	Describe the processes used to decide which studies were eligible for each synthesis (e.g., tabulating the study intervention characteristics and comparing against the planned groups for each synthesis (item 5)).	Figure 1
	13b	Describe any methods required to prepare the data for presentation or synthesis, such as handling of missing summary statistics, or data conversions.	Not applicable
	13c	Describe any methods used to tabulate or visually display results of individual studies and syntheses.	Tables 1 and 2
	13d	Describe any methods used to synthesize results and provide a rationale for the choice(s). If meta-analysis was performed, describe the model(s), method(s) to identify the presence and extent of statistical heterogeneity, and software package(s) used.	Tables 1 and 2, Figure 1
	13e	Describe any methods used to explore possible causes of heterogeneity among study results (e.g., subgroup analysis, meta-regression).	Section 2.4
	13f	Describe any sensitivity analyses conducted to assess robustness of the synthesized results.	Not applicable
**Reporting bias assessment**	14	Describe any methods used to assess risk of bias due to missing results in a synthesis (arising from reporting biases).	Tables 3-5
**Certainty assessment**	15	Describe any methods used to assess certainty (or confidence) in the body of evidence for an outcome.	Tables 3-5

**RESULTS**			
**Study selection**	16a	Describe the results of the search and selection process, from the number of records identified in the search to the number of studies included in the review, ideally using a flow diagram.	Section 3.1 and Figure 1
	16b	Cite studies that might appear to meet the inclusion criteria, but which were excluded, and explain why they were excluded.	Line 294–299 and Figure 1
**Study characteristics**	17	Cite each included study and present its characteristics.	Tables 1 and 2
**Risk of bias in studies**	18	Present assessments of risk of bias for each included study.	Tables 3-5
**Results of individual studies**	19	For all outcomes, present, for each study: (a) summary statistics for each group (where appropriate) and (b) an effect estimate and its precision (e.g., confidence/credible interval), ideally using structured tables or plots.	Tables 3-5
**Results of syntheses**	20a	For each synthesis, briefly summarize the characteristics and risk of bias among contributing studies.	Sections 3.3 and 3.4
	20b	Present results of all statistical syntheses conducted. If meta-analysis was conducted, present for each the summary estimate and its precision (e.g., confidence/credible interval) and measures of statistical heterogeneity. If comparing groups, describe the direction of the effect.	Tables 3-5
	20c	Present results of all investigations of possible causes of heterogeneity among study results.	Sections 3.2–3.4
	20d	Present results of all sensitivity analyses conducted to assess the robustness of the synthesized results.	Not applicable
**Reporting biases**	21	Present assessments of risk of bias due to missing results (arising from reporting biases) for each synthesis assessed.	Tables 3-5
**Certainty of evidence**	22	Present assessments of certainty (or confidence) in the body of evidence for each outcome assessed.	Not applicable
**DISCUSSION**			
**Discussion**	23a	Provide a general interpretation of the results in the context of other evidence.	Line 300–373
	23b	Discuss any limitations of the evidence included in the review.	Line 384–387
	23c	Discuss any limitations of the review processes used.	Line 376–388

	23d	Discuss implications of the results for practice, policy, and future research.	Line 399–408
**OTHER INFORMATION**			
**Registration and protocol**	24a	Provide registration information for the review, including register name and registration number, or state that the review was not registered.	CRD42025631901
	24b	Indicate where the review protocol can be accessed, or state that a protocol was not prepared.	Methods section, Figure 1
	24c	Describe and explain any amendments to information provided at registration or in the protocol.	Not applicable
**Support**	25	Describe sources of financial or non-financial support for the review, and the role of the funders or sponsors in the review.	Funding section, Line 423–425
**Competing interests**	26	Declare any competing interests of review authors.	Conflicts of Interest section, Line 430–432
**Availability of data, code and other materials**	27	Report which of the following are publicly available and where they can be found: template data collection forms; data extracted from included studies; data used for all analyses; analytic code; any other materials used in the review.	Figure 1, Tables 1–5, S1, S2 and S3

Table S2. PRISMA 2020 abstract checklist.

Topic	No.	Item	Reported?
**TITLE**			
**Title**	1	Identify the report as a systematic review.	Yes
**BACKGROUND**			
**Objectives**	2	Provide an explicit statement of the main objective(s) or question(s) the review addresses.	Yes
**METHODS**			
**Eligibility criteria**	3	Specify the inclusion and exclusion criteria for the review.	No
**Information sources**	4	Specify the information sources (e.g., databases, registers) used to identify studies and the date when each was last searched.	Yes
**Risk of bias**	5	Specify the methods used to assess risk of bias in the included studies.	Yes
**Synthesis of results**	6	Specify the methods used to present and synthesize results.	No

**RESULTS**			
**Included studies**	7	Give the total number of included studies and participants and summarize relevant characteristics of studies.	Yes
**Synthesis of results**	8	Present results for main outcomes, preferably indicating the number of included studies and participants for each. If meta-analysis was conducted, report the summary estimate and confidence/credible interval. If comparing groups, indicate the direction of the effect (i.e., which group is favored).	Yes
**DISCUSSION**			
**Limitations of evidence**	9	Provide a brief summary of the limitations of the evidence included in the review (e.g., study risk of bias, inconsistency and imprecision).	No
**Interpretation**	10	Provide a general interpretation of the results and important implications.	Yes
**OTHER**			
**Funding**	11	Specify the primary source of funding for the review.	No
**Registration**	12	Provide the register name and registration number.	No

From [21]: Page, M.J.; McKenzie, J.E.; Bossuyt, P.M.; Boutron, I.; Hoffmann, T.C.; Mulrow, C.D.; Shamseer, L.; Tetzlaff, J.M.; Akl, E.A.; Brennan, S.E.; et al. The PRISMA 2020 statement: An updated guideline for reporting systematic reviews. *MetaArXiv* 2020. September 14. https://doi.org/10.31222/osf.io/v7gm2. (accessed on 21 February 2025). For more information, visit: www.prisma-statement.org.

Table S3: Summary of overall score/maximum score of the included studies. Quality assessment of studies was performed with a quality checklist tool (Cosme et al. [23]). Association between leptin levels and bone mineral density (BMD) were represented as negative association (yellow), positive association (green), and no association (gray).

**Reference**	**Overall score/ Maximum score**	**Association between leptin levels and BMD**
Ormarsdóttir et al. 2001 [24]	82 %	yellow
Sato et al. 2001 [25]	82 %	yellow
Huang et al. 2004 [26]	75 %	gray
Papadopoulou et al. 2004 [27]	75 %	green
Javaid et al. 2005 [28]	93 %	green
Oh et al. 2005 [29]	86 %	yellow
Yaris et al. 2005 [30]	79 %	gray
Crabbe et al. 2006 [31]	86 %	green
Lorentzon et al. 2006 [32]	86 %	yellow
Qiu et al. 2007 [33]	89 %	green
Söderpalm et al. 2007 [34]	86 %	yellow
Peng et al. 2008 [35]	86 %	gray
Vondracek et al. 2009 [36]	96 %	green

Ghonemy et al. 2011 [37]	86 %	
Koutroubakis et al. 2011 [38]	96 %	
Sienkiewicz et al. 2011 [39]	100 %	
Fountoulis et al. 2012 [40]	93 %	
Anagnostis et al. 2013 [41]	86 %	
Brown et al. 2013 [42]	82 %	
Veselá et al. 2016 [43]	89 %	
Ho-Pham et al. 2017 [44]	86 %	
Krishnan et al. 2022 [45]	96 %	
Normand et al. 2022 [46]	93 %	
Bonnet et al. 2005 [47]	93 %	
Martin et al. 2005 [48]	100 %	
Martin et al. 2007 [49]	96 %	
Motyl et al. 2009 [50]	96 %	
Stunes et al. 2012 [51]	93 %	
